# Barriers and Facilitators to Dental Care Services Utilization Among Children With Disabilities: A Systematic Review and Thematic Synthesis

**DOI:** 10.1111/hex.70049

**Published:** 2024-09-30

**Authors:** Shiamaa Al‐Mashhadani, Mona Nasser, Anas Alsalami, Lorna Burns, Martha Paisi

**Affiliations:** ^1^ Peninsula Dental School University of Plymouth Plymouth UK; ^2^ General Dental Department, Dubai Dental Hospital Dubai Health Dubai UAE; ^3^ Peninsula Dental School, Faculty of Health University of Plymouth Plymouth UK; ^4^ Hamdan Bin Mohammed College of Dental Medicine, Mohammed Bin Rashid University of Medicine and Health Sciences Dubai Health Dubai UAE; ^5^ School of Nursing and Midwifery University of Plymouth‐SNAM Plymouth UK

**Keywords:** barriers and facilitators, children with disabilities, dental care services, holistic approach, multidisciplinary collaboration, oral health promotion, stigma

## Abstract

**Background:**

This systematic review investigates barriers and enablers to dental care utilization by disabled children. Given the high global prevalence of disabilities in children, coupled with poor oral hygiene and a 45% rate of dental caries in this group, developing inclusive oral health strategies is critical. The review aims to synthesize literature on factors affecting oral healthcare improvement for disabled children, identifying barriers, facilitators and knowledge gaps.

**Methods:**

The review was conducted following the Joanna Briggs Institute's methods and reported according to PRISMA guidelines. A comprehensive search spanned multiple databases, considering perspectives from carers, parents, dentists and health professionals. The focus was on studies involving children up to age 17 with disabilities, as defined by WHO, using dental care services. Exclusions included non‐qualitative studies, populations over 18 and nondisabled children. There were no restrictions on publication date or language. Thematic synthesis of the studies extracted themes related to barriers and enablers in oral healthcare for disabled children.

**Results:**

Thematic synthesis identified five overarching themes: stigma, communication issues, professional development, oral health education and medical‐dental collaboration. Facilitators included enhancing accessibility and availability of dental care through a holistic approach, improving dental care facility environments and ensuring skilled dental care providers.

**Discussion:**

The review underscores the importance of interprofessional collaboration, improved parent/caregiver education and specialized dental facilities to support children with disabilities. It identifies key barriers and facilitators in dental care, including challenging stereotypes, improving communication between providers and parents, enhancing holistic training and addressing gaps in oral health education and integrated healthcare systems.

**Conclusion:**

Addressing the complex dynamics of dental care for disabled children is essential for developing inclusive and effective preventive and therapeutic strategies. This review highlights the need for tailored approaches and enhanced support systems to improve oral health outcomes in this vulnerable population.

**Patient and Public Contribution:**

The members of the family support department, Middle East and North Africa (MENA) Organization for Rare Disease and Disability who provided the disability voice and contributed to providing input to the review protocol.

## 1 Introduction

Child oral health is a significant global public health concern, especially among children with disabilities who show a higher susceptibility to dental issues compared to their typically developing peers [1, 2, 3]. The World Health Organization (WHO) defines disability using the International Classification of Functioning, Disability and Health (ICF). According to the ICF, it reflects the interaction between an individual's health condition and contextual factors such as environmental and personal factors [1]. Disabilities among children globally vary widely and each presents unique challenges and requires tailored interventions and support systems to ensure their well‐being and inclusion in society.

These conditions necessitate complex, multi‐faceted interventions that address not only the specific impairments but also the broader social, educational and environmental factors that impact the child's quality of life. Effective intervention strategies often involve a multidisciplinary approach, incorporating medical treatment, therapeutic services, educational accommodations and family support [4]. The preservation of proper oral hygiene is a critical component within this complexity, as children with disabilities often face significant barriers to accessing and receiving adequate oral healthcare for children with disabilities, such as cerebral palsy, Down syndrome and autism, as reported by the National Institute of Dental and Craniofacial Research [5]. These barriers can include physical limitations that hinder dental hygiene practices, sensory sensitivities that require adjustments to the process and environment of the dental practice and cognitive impairments that necessitate specialized communication and care techniques. Communication difficulties may hinder the clear expression of oral health needs, overlooking essential aspects of dental care. Furthermore, the absence of specialized dental services customized to address the unique needs of these children may increase oral health complications [6]. Oral health in children with disabilities may be negatively impacted by medications, which may increase their vulnerability to dental problems or induce xerostomia [7].

Dietary issues, such as restricted diets or sensory sensitivities, may further worsen disparities in oral health within this demographic. A more nuanced approach would provide a comprehensive understanding of the challenges faced by parents and caregivers in maintaining oral health for children with disabilities. It is important to recognize that interventions may be postponed due to a combination of socio‐economic, cultural and personal factors. Limited financial resources, cultural beliefs, transportation issues and personal struggles can make it difficult for parents to prioritize preventive oral care and access necessary oral health services. Understanding these complexities is crucial in supporting parents and caregivers in improving their children's oral health [8, 9].

Furthermore, these children may encounter challenges in achieving optimal oral health, such as physical barriers like limited access to dental facilities and a lack of supportive resources. Oral health outcomes for children with disabilities may be adversely affected by disparities in policies regarding oral health and deficiencies in educational resources for caregivers, parents and healthcare professionals. Understanding the intricate dynamics among the factors contributing to differences in child oral health is essential for developing comprehensive preventive and therapeutic strategies [10].

Ensuring consistent and preventive oral healthcare is essential for the overall health of children with disabilities. The importance of addressing oral health issues in children with disabilities is emphasized throughout research studies globally. This involves recognizing that children with visual and auditory impairments, cerebral palsy, Down syndrome and other disabilities require specialized oral healthcare [2, 3]. Identifying risks for children with special healthcare needs is essential to educate health providers on identifying and overcoming obstacles to dental care for this high‐risk population [4]. Moreover, research underscores the importance of identifying a dentist for children with disabilities and suggests that their first dental appointment be scheduled before age one. This is significant in children with special requirements [11]. Addressing these unique oral healthcare needs requires the collaboration of dental professionals with other healthcare providers, caregivers and educators to develop comprehensive, individualized care plans [5, 6, 7].

This study aims to understand the barriers and facilitators affecting the utilization of dental healthcare in children with disabilities and to identify gaps in knowledge that can help enhance oral health in this population.

## 2 Methodology

This qualitative systematic review followed core methods for evidence synthesis provided by the Joanna Briggs Institute methods for evidence synthesis [8]. The review question was structured using the PerSPecTIF framework [9]. The PerSPecTIF framework helped to design a question that reflects the multifaceted nature of the problem (Table 1), ensuring that all relevant aspects are considered including the understanding of the perspectives of the stakeholders, context (e.g., settings, environment) and other elements such as time and place.

**Table 1:** PerSPecTIF framework used for the review question.

Perspective (Per)	Setting (S)	Phenomenon of interest (P)	Environment (e)	Comparison (c)	Time (TI)	Findings (F)
From the perspective of carers, parents dental and non‐dental health professionals	Oral healthcare settings and the home environment where oral healthcare occurs	The current practices, advice and support provided by the dental care system	Within the broader health environment, including policies, accessibility and support systems	The standard dental practice did not change or adjust for children with disability	Any point in time that an oral health, encounter or behaviour happens	In relation to barriers and facilitators that affect the oral healthcare of children with disability

The thematic synthesis followed the approach outlined by Harden and Thomas [10]. The protocol for the systematic review was published and registered with PROSPERO.

### 2.1 Eligibility Criteria

The eligibility criteria were designed to include the perspectives of carers, parents, dentists and health professionals. Studies were included if they involved children with disabilities, as defined by the WHO. WHO uses a wide definition of disability that recognizes the complex interaction between an individual's health condition, such as cerebral palsy and Down syndrome and contextual factors, including environmental and personal factors. Disabilities considered were physical, sensory, intellectual and developmental impairments and focused on their use of dental care services. Children aged 17 years and under were eligible for inclusion. Studies were excluded if they did not utilize qualitative data collection and analysis methods, involved a population over 18 years old or included children without disabilities. There were no restrictions on publication date, language or country of publication.

Various study designs, including literature reviews and empirical research, were considered to understand the barriers, facilitators and interventions in oral health for children with disabilities. This included governance, health systems, dental and medical health providers, caregivers as well as clinical or preventive interventions.

### 2.2 Search Strategy and Information Source

A comprehensive search was conducted on Embase, MEDLINE, CINAHL, DOSS, SocINDEX and PsycINFO databases. Grey literature [12] was identified by an internet search using the search engine Google, established sources of grey literature and websites of relevant organizations, including FDI, WHO, International Association of Disability and Oral Health and British Society of Disability and Oral Health. Database and grey literature searches were last updated in March 2023. This evaluation consisted of three stages: database search, inspection of titles and abstracts and full‐text analysis.

### 2.3 Search Strategy

The search strategies were co‐designed with an information specialist using a combination of subject headings. The searches comprised two blocks to represent the concepts of disabilities and dental or oral health. Subject headings and syntax were translated across database interfaces. The full search strategies are detailed in Appendix A.

### 2.4 Selection Process

Primary studies that used qualitative methods for data collection (such as interviews and observations) and/or used qualitative methods for data analysis (e.g., thematic analysis) were included in the data collection. Studies that did not use qualitative analysis (e.g., descriptive analysis) were excluded even if the study was qualitative. Search results were uploaded for screening to Rayyan [13], a systematic review web application. Two reviewers independently screened all titles and abstracts against the inclusion and exclusion criteria. Full texts of potentially relevant articles were retrieved and independently screened by the two reviewers. Where the two reviewers disagreed on article inclusion, consensus was reached by discussion with the third reviewer.

### 2.5 Data Extraction

The data items retrieved from the qualitative studies included the title and citation of the article, objective, aim, type of study and study design. It also included information on barriers, facilitators, access and provision to dental care and children's behaviours towards dental visits.

### 2.6 Data Synthesis and Analysis

Two reviewers employed thematic synthesis to undertake an inductive analysis of the qualitative data from the investigations [10]. The data were coded, and codes with similar meanings or experiences were grouped. The most prominent, recurring or important literary descriptive themes were recognized. The constant comparative approach [14] was employed to ensure concept translation from one study to the next, looking for similarities and contrasts in the findings provided in the papers/reports. Analytic themes were developed by investigating whether the various descriptive themes resulted in a novel interpretation of the data as ‘third order interpretation’. The findings follow the PRISMA guidelines, ensuring transparency and reproducibility.

## 3 Results

**Figure 1:**
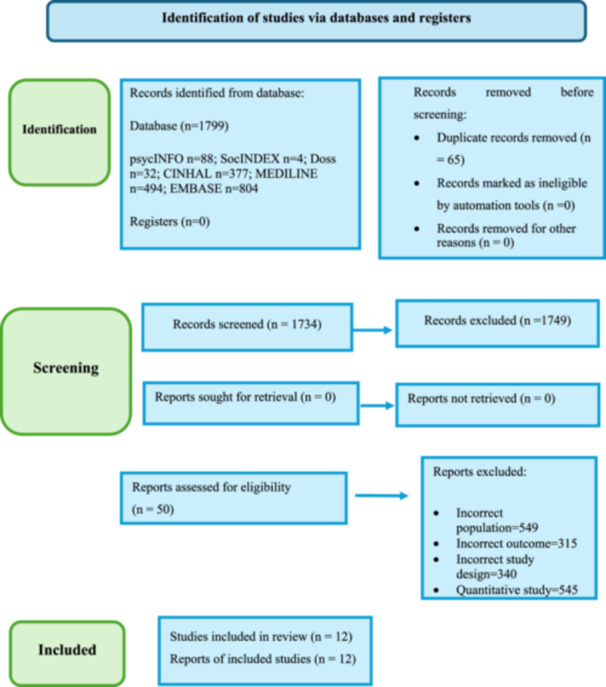
PRISMA flowchart of the dental care services utilization among children with disabilities.

The flow of information is presented in the PRISMA diagram in Figure 1. Twelve peer‐reviewed publications were found through the inclusion criteria. Four publications were qualitative interviews with parents and their disabled children; three publications were qualitative interviews with both parents and dental providers, three studies had health providers and parents interviewed and two studies had health providers interviewed. The research was carried out in several countries across Europe, Asia, America and Brazil. Participants were recruited from healthcare institutions, schools and oral health promotion and prevention programmes and interviewed individually or in focus groups. Grey literature was searched as mentioned in Appendix A. However, no specific aspects focusing on barriers and facilitators for oral healthcare of disabled children were identified. The information was more generalized and focused on the disabled population in general.

### 3.1 Barriers and Facilitators to Dental Care Services Utilization Among Children With Disabilities

Five analytical themes were identified from the analysis of the descriptive data reporting the perspective and experiences of the parent/carer or dental and non‐dental care professionals and are presented in Table 2. A full description of all the studies is provided in Appendix B.

**Table 2:** Themes identified from the synthesis of the descriptive themes.

Analytical themes	Descriptive themes extracted from the studies related to parents and children with disabilities, dental and non‐dental health providers
Challenging stereotypes in dental care for individuals with disabilities	Stigma towards the disability The need for respect and autonomy Gender stereotyping of the ability to handle children with disabilities Negative influence of parent's past experiences [10, 12]
Navigating the communication channels between dental providers and parents of children with disabilities	Miscommunication of information between health providers and parents Lack of a pre‐communication system to transfer information Development and training [14, 15]
Holistic training to address gaps in the dental management of disabled	A need for special care dentistry Mentorship programmes Setting the dental practice environment to serve special needs Training GDP to handle children's needs and complex medical problems Lack of behaviour management skills [15, 16]
Addressing gaps in oral health education and resource accessibility	Lack of reliable resources Lack of oral hygiene knowledge Lack of access to preventive programmes Tailor‐made materials to educate parents Taking the initiative to innovative ways for promotion [17, 18]
Difficulties in navigating and improving integrated oral healthcare systems	Unclear referral system Negative influence of parent's past experiences Lack of a clear roadmap to follow for parents Lack of multidisciplinary teams and collaboration Lack of medical health providers' development skill training [15, 18]

### 3.2 Challenging Stereotypes in Dental Care for Individuals With Disabilities

From the analysis of three studies conducted by Fadel et al. [17], Oliveira et al. [19] and Wibisono et al. [20], societal stigma towards children with disabilities and their families was the main theme. In addition, stereotyping female dental providers as more capable of handling children with disabilities than male providers was one of the relevant codes for this theme. All three studies discussed that stigmatizing attitudes of health professionals towards children with disabilities in terms of managing them during dental care is a barrier for persons with disabilities in accessing high‐quality healthcare services [15] and for health providers [21].

The main reasons for stigma towards treating children with disabilities in the three studies were the fear of the unknown, complexity of medical conditions, lack of knowledge of the different conditions of disability, behaviour attitudes of the patients and financial compensation of time and efforts provided by the dental providers.

### 3.3 Navigating the Communication Channels Between Dental Providers and Parents of Children With Disabilities

In this theme, Fadel et al., Alves et al., Hallberg et al. and Junnerker et al. focused on communication issues related to children and their families having difficulty accessing dental healthcare. The studies showed that having a disability might impede communication between health providers and patients [16]. Fadel et al., Alves et al., Hallberg et al., Junnerker et al. and Lim et al. [13, 17, 18, 22] mentioned the need for communication between the dental providers and the children and parents. Hallberg et al. and Alves et al. mentioned a missing aspect of a transparent system to exchange information on the child's condition, their triggers and any underlying disability that was not informed previously. They wanted a more transparent and more efficient way to have enough information on their patient to help them better plan, diagnose and treat their patient. Alves et al emphasized the role of parents in communication as many of them were not enthusiastic about offering information on their children and had to rely on their observational skills to understand the children better. The issues of communication mentioned by Hallberg et al. and Lim et al. were a two‐way process: information from the patients to the dental provider, the dental supporting staff and also from the dental provider towards the parents and child.

#### 3.3.1 Holistic Training to Address Gaps in Dental Management of Disabled

Training for dental providers mentioned by Fadel et al. was related to enhancing dental experience in child management through exposure via campaigns [17], Hallberg et al., Lang Junnarkar et al. and Klingberg et al. mentioned gaps in professional training for dental practitioners [13, 22, 23, 24] and lack of enough training on behaviour management skills [18]. Suggestions by Lim et al. were given to have opportunities provided to the dentist who was enthusiastic and willing to learn more about care for children with disabilities and emphasis on expanding the professional experience to general dental practitioners to increase access to dental care [25, 26]. The study by Fadel et al. mentioned that the participants of the preventive programme were asking for more opportunities similar to the one provided to allow the dental providers to gain skills to help them provide quality care for disabled children. Another result was the positive attitudes towards caring for persons with disabilities. Lim et al. in his study mentioned that the dentists interviewed in the study suggested that there should be opportunities for training and development for the dental providers who ‘might be in there because they actually want to make a difference…something intrinsic in that person's internal value system about what makes their life worthwhile’ [25]. The study by Lim et al. also highlighted the necessity for mentorship programmes, providing dental providers with direct access to specialists or experts in behaviour management, clinical knowledge and the management of various disabilities [25].

#### 3.3.2 Addressing Gaps in Oral Health Education and Resource Accessibility

We identified four studies [17, 19, 20, 27] underscoring the significance of preventive and oral education programmes in reducing dental diseases. The focus was on oral health education directed at parents or caregivers to address the specific needs of children, emphasizing the importance of customizing educational materials according to each child's requirements. The emphasis extended to sustainable programmes rather than one‐time actions, aiming to maximize the benefits of any preventive or educational approach. Furthermore, there was advocacy for taking the initiative in discovering innovative methods and employing diverse materials and media to accommodate various learning levels and abilities for both parents and children [21, 28, 29].

#### 3.3.3 Difficulties in Navigating and Improving Integrated Oral Healthcare Systems

In our review, Lang et al., Stein Duker et al., Junnarkar et al., Klingberg et al. and Owens [13, 23, 24, 27, 30] highlighted the absence or ambiguity surrounding the access to multidisciplinary teams in dental and medical care and the inadequacy of a well‐defined referral system to connect with other health providers. The studies distinctly articulate the need for a comprehensive roadmap, urging health providers to establish connections, foster collaboration and exchange essential information for the child's health journey.

## 4 Discussion

This systematic review looks into existing literature to examine the factors affecting the oral healthcare of children with disabilities from a qualitative perspective. The review resulted in five themes related to stereotypes in dental care, communication between dental providers and parents, training to address gaps in dental management, sustainable oral health education and establishing dental‐medical collaboration.

### 4.1 Challenging Stereotypes in Dental Care for Individuals With Disabilities

The review identified factors pertaining to caregivers and parents of children with disabilities, notably linked to societal stigma and challenges within the health system [13, 17, 18, 19]. Dental providers displayed reluctance in treating these patients, citing reasons such as insufficient dental skills [13, 17, 18, 22, 23, 25], inadequate compensation for their efforts [18] and a scarcity of dentists willing to provide care [24, 25]. Regarding factors associated with dental professionals delivering care, one study examined the stereotype that female dental providers are more adept at handling children with disabilities [17]. This perception could be influenced by cultural norms associating females with maternal roles or male dentists' reported lack of interest in such cases [17]. Parents stressed the significance of being treated with respect and autonomy [18], emphasizing the need for clear communication between them, the children and dental providers [13, 17, 23]. There is a need for dental educational institutions to train graduates to be more competent in managing a diverse patient population and to have the interpersonal and communication skills necessary to function successfully in a diverse work environment. This issue should be addressed immediately as it may foster a ‘we versus they’ attitude that can be divisive or disrespectful, hindering efforts to embrace diversity as a valuable aspect of society.

International policies support children's rights and require that inclusion and fair care should be provided to them. Health professionals have a crucial role in promoting inclusion in healthcare services. It is critical to analyse health professionals' stigmatizing attitudes and raise awareness further to increase inclusion in mainstream healthcare services.

These points should be addressed, and a better understanding of how to overcome these obstacles could help and support an environment where dental providers are less likely to shy away from treating or caring for disabled children, therefore resulting in a less stigmatized attitude towards this valuable population.

### 4.2 Navigating the Communication Channels Between Dental Providers and Parents of Children With Disabilities

Many studies underscored the prevalence of miscommunication, highlighting the necessity for a structured exchange of information and discussions, crucial for fostering positive relationships between parents and dentists [13, 17, 23]. Conversely, parents' negative past experiences with dental care significantly impacted their children's interactions with dental providers [17, 18, 23], potentially due to limited access to reliable information and resources for enhancing their understanding of dental and oral healthcare [13, 23].

Delivering patient‐centred care is dependent on efficient provider–patient communication, which ensures a complete understanding and address of patients' needs and preferences.

Communication between health providers and parents of children with disabilities is an essential factor in providing desirable outcomes and results [22].

In a study conducted by Salmasi et al., inadequate communication occurred between the dental professional and the patients who had disabilities or their caregivers. This elicited emotional responses such as frustration, feeling left out and unable to contribute to the treatment plan [24].

Balkaran et al. expressed a similar concern in their research. One of the caregivers interviewed emphasized the need for improved communication. They wished for dental providers to enhance their communication skills and acquire methods to engage with disabled individuals effectively. For instance, the suggestion included having someone within the practice who understands sign language [25].

In addition, dental providers can use simple measures to address communication barriers for people with different disabilities. Given that many patients may be unable to communicate verbally, dental X‐rays are frequently used to diagnose dental abnormalities such as caries. Patient trust is essential, as are systematic and empathic diagnostic procedures. All dental practice staff members, including front desk personnel, hygienists and dental assistants, must be trained to communicate and connect successfully with these patients, fostering enhanced comfort and collaboration. Recognizing the unique nature of each individual with disabilities and their various needs, dentists can customize treatment plans by engaging in discussions with carers and adapting care to address specific issues for each person [26].

Effective communication emerged as a crucial factor, with dental providers stressing the importance of understanding the child and parents' circumstances and seeking comprehensive information on the child's condition before appointments to facilitate more accurate treatment planning [13]. It is considered crucial in the context of oral healthcare for children with disabilities. Delivering patient‐centred care is dependent on efficient provider–patient communication, which ensures a complete understanding and address of patients' needs and preferences.

Communication between health providers and parents of children with disabilities is an essential factor in providing desirable outcomes and results [22].

In a study conducted by Salmasi et al., inadequate communication occurred between the dental professional and the patients who had disabilities or their caregivers. This elicited emotional responses such as frustration, feeling left out and unable to contribute to the treatment plan [24]. Balkaran et al. expressed a similar concern in their research. One of the caregivers interviewed emphasized the need for improved communication. They wished for dental providers to enhance their communication skills and acquire methods to engage with disabled individuals effectively. For instance, the suggestion included having someone within the practice who understands sign language [25].

In addition, dental providers can use simple measures to address communication barriers for people with different disabilities. Given that many patients may be unable to communicate verbally, dental X‐rays are frequently used to diagnose dental abnormalities such as caries. Patient trust is essential, as are systematic and empathic diagnostic procedures. All dental practice staff members, including front desk personnel, hygienists and dental assistants, must be trained to communicate and connect successfully with these patients, fostering enhanced comfort and collaboration. Recognizing the unique nature of each individual with disabilities and their various needs, dentists can customize treatment plans by engaging in discussions with carers and adapting care to address specific issues for each person [26].

This proactive approach to communication is seen as instrumental in facilitating more accurate treatment planning. Essentially, it underscores the importance of a thorough and informed communication process between dental providers, children with disabilities and their parents for optimal oral healthcare outcomes.

### 4.3 Holistic Training to Address Gaps in the Dental Management of Disabled

A significant aspect highlighted in the reviewed studies and existing literature was the imperative need to develop and train dental providers with appropriate skills to manage and treat patients with disabilities. Recommendations extend beyond acknowledging this barrier to proposing a structured training system that encompasses behaviour management, handling complex medical conditions, incorporating undergraduate training and exposure to relevant cases.

Theirer and Meyerowitz, in their study, concluded that more education and training in special care dentistry will lead to better‐educated dentists and the desired result of better access to care for special needs patients [27]. In addition, Smith et al. provided similar results to our review that covered the main barriers to training dental providers: the healthcare providers’ lack of knowledge or knowledge gained through targeted interventions.

This would improve healthcare professionals' self‐efficacy in response to intervention for the children. Persistent difficulties were communicating with or about individuals with disabilities that require training and gaining skills for communication, as mentioned earlier, and the sense that few disabilities targeted health resources existed, which caused a barrier to gaining extra knowledge for the dental team [20]. Such programmes aim to enhance access to more proficient dentists capable of caring for children.

#### 4.3.1 Fostering Sustainable Oral Health Education for Parents and Children With Disabilities

Another facilitator for improving oral health was identified in the availability of resources for both patients and dental providers. Resources were categorized into reliable tools for dental providers to utilize during treatment and preventive interventions, as well as materials for educating parents and patients to enhance and maintain optimal oral health. Innovative methods, such as leveraging media and technology and collaboration with professionals like psychologists, were proposed in one study to effectively communicate advice and knowledge to patients.

Numerous studies have examined the measurable enhancement in knowledge following the provision of dental health education to parents and children with disabilities. Additionally, these studies have assessed the sustained effectiveness of dental health education programmes in elevating knowledge levels within this population over the long term. Research findings also proposed that consistent reinforcement through repeated oral health education sessions led to a significant enhancement in knowledge about oral health, improved practices and reduced oral health issues. Targeted areas and environments, such as schools that had regular exposure to dental health education programmes, demonstrated better scores in various aspects of oral health conditions, including plaque index, in comparison to schools with less frequent exposures [28].

Oral health education should target both the caregiver/parent and the child. A caregiver's increased knowledge about oral health contributes to a more positive attitude, fostering healthier habits. The acquired knowledge directly influences attitudes, leading to behavioural changes. Caregivers with a higher knowledge level are more likely to actively participate in their children's oral hygiene routine, elevating their overall attitude. This positive attitude, in turn, encourages a more frequent engagement in oral hygiene for the child [21].

Conversely, maintaining oral hygiene and care is a collaborative family endeavour that should seamlessly integrate into the family routine. Tailored oral health education, specifically designed to cater to the child's abilities, becomes necessary. Whether a child is inclined towards visual or auditory learning or a show, tell, do approach, this should be contemplated during the planning, development and delivery of educational materials. For children with disabilities, a personalized approach is essential, recognizing that there is no one‐size‐fits‐all solution. Each child requires a unique strategy aligned with their timeframe and follow‐up requirements. Embracing such an approach proves to be more cost‐effective and time‐efficient and yields superior results. These resources must be readily available to families and children, ensuring accessibility. The advancement of technology and media offers a myriad of options that oral health promoters can utilize for the benefit of children. These approaches should be actively employed and promoted during oral health campaigns and within healthcare settings.

### 4.4 Establishing a Clear Path for Dental‐Medical Collaboration

Lastly, the role of the healthcare system in enhancing access and care for children with disabilities was emphasized in multiple studies. Establishing a robust communication system between dental and medical care providers and parents was deemed essential for comprehensive patient understanding. The system should incorporate a clear referral pathway to ensure timely care without bureaucratic delay [13, 27].

Collaborative efforts with various entities, particularly health and social care professionals, generate significant results by creating networks and service pathways. Communication between the dental team and other disciplines is most effective when the dental team participates in interdisciplinary assessments and care processes. The collaborative care planning process provides an excellent chance to integrate and emphasize the importance of dental healthcare in terms of overall health and well‐being [30].

The inclusion of nutrition specialists, speech and language therapists, specialist nurses, occupational therapists, psychologists, genetic counsellors and oral health providers in interdisciplinary teams can significantly enhance the support provided to oral health professionals. This collaboration goes a long way in promoting and identifying dental issues, thereby increasing opportunities for access to dental care. The American Academy of Developmental Medicine and Dentistry (AADMD) provides a robust example of collaborative efforts in dental and medical fields to tackle health disparities in specific populations. Their community‐based fellowships in developmental dentistry and medicine offer a potential solution to address crucial training, service and research needs simultaneously [31].

This request underscores the necessity to concentrate on actionable steps to enhance the experiences of children and their families within healthcare systems. Addressing oral health problems necessitates collaborative efforts at academic, community and policy levels to incorporate oral health into comprehensive healthcare seamlessly. Establishing partnerships that bring together dental, medical and other health professionals and trainees is essential, particularly in benefiting the health of vulnerable populations, such as children with disabilities. Given the historical oversight of oral health, it becomes imperative for all health professionals to convey unified messages, ensuring that disabled children and their families can foster lifelong oral health. Essentially, oral health should be seamlessly integrated into overall health, and all healthcare providers play a role in its promotion. Achieving this goal requires comprehensive initiatives that simultaneously embed oral health promotion into medical care.

Additionally, dental providers and their teams must be trained in the skills required to treat children with different disabilities effectively. Specialized dental programmes and mentorship initiatives were also recommended for professional development and improved access to dental providers [25]. Educational efforts should extend to creating a practice environment sensitive to the triggers of vulnerable patients, involving training for all personnel in contact with the child and their families [31]. This holistic approach aims to foster an ideal environment for dental care and establish connections among various disciplines of care through multidisciplinary teams, ultimately enhancing the overall health of the child.

**Figure 2:**
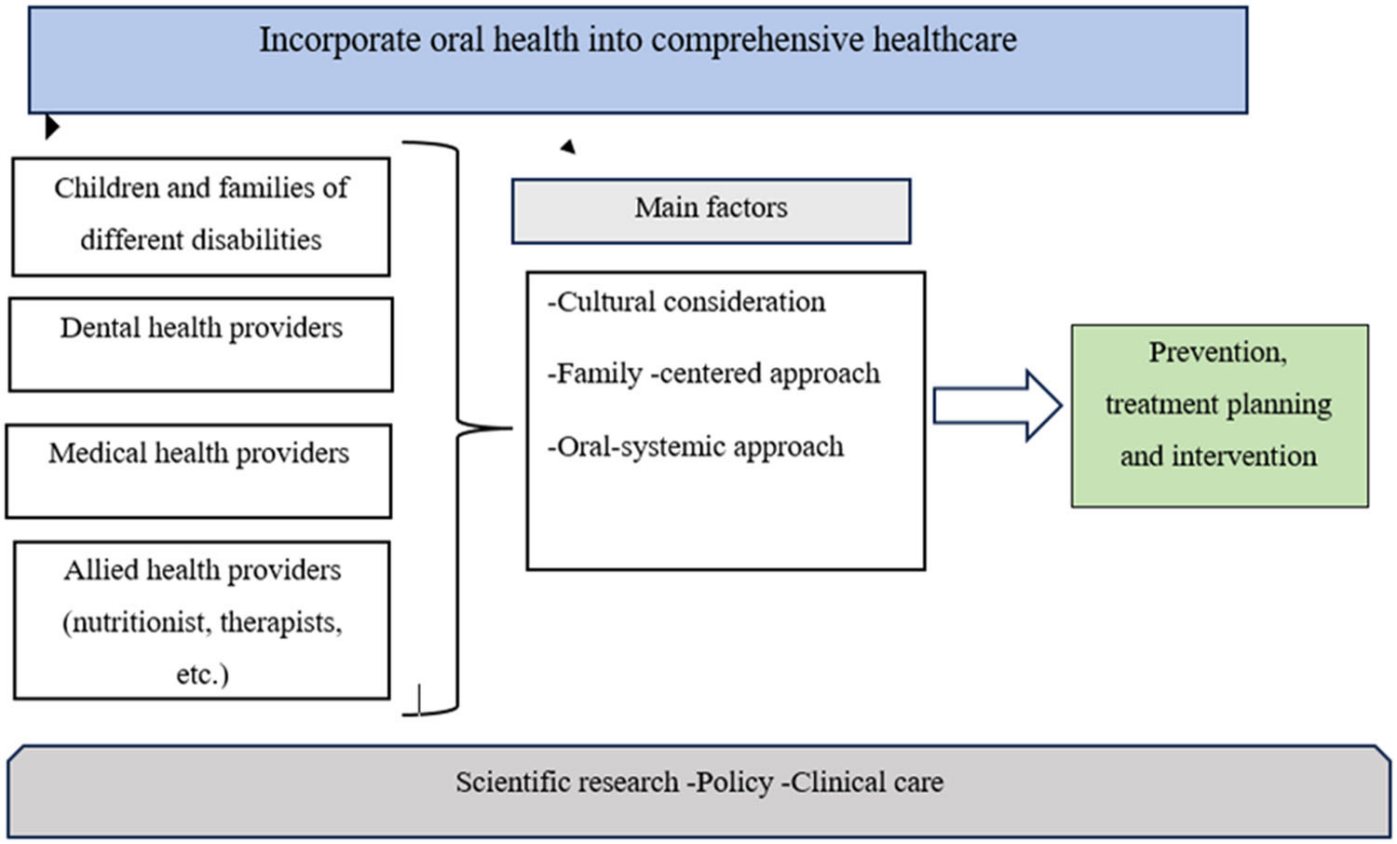
A joint roadmap that includes both preventive and treatment planning setup.

Success in the above relies on considering the cultural context, oral systemic health and a family‐centred approach. An example of such an approach is a collaborative policy agreement between paediatric dentists and paediatricians to work on a comprehensive and holistic approach to caring for the children; there should be a joint roadmap that includes both a joint preventive and treatment planning setup (Figure 2).

## 5 Review Author's Reflexivity

The perspectives of the review authors regarding barriers and facilitators, utilization of dental care and other factors may affect the manner in which the collection, synthesis and interpretation of the data was done. All the review authors believed that understanding the barriers and facilitators from the perspective of different stakeholders in a child's life was valuable to improve the oral health of children with disabilities, but that important barriers exist to allow that. To minimize the risk that our perspectives as authors influence the synthesis and interpretation, we discussed the contradictory findings. We accounted for these differences, and any other issues that may have contributed to the interpretation of the review findings, by describing it in a ‘Reflexivity’ section when publishing the full review.

## 6 Strengths and Limitations

Utilizing a qualitative methodology, this systematic review extensively explores the barriers and enablers affecting the enhancement of oral healthcare for children with disabilities. A pivotal connection of the elements is established through analysing the factors impacting oral health and acknowledging the engagement of stakeholders in the child's life. This approach enriches our understanding and presents comprehensive solutions with the potential to surmount the identified barriers. Furthermore, the study introduces a model that contributes to a deeper understanding of an important factor identified in the review—namely, establishing a clear pathway for dental‐medical collaboration.

One noteworthy limitation of this study pertains to the comprehensive screening process applied to select relevant studies. While this rigorous approach aimed to ensure the inclusion of high‐quality and pertinent research, it inadvertently restricted the number of studies incorporated. While instrumental in maintaining the study's robustness, the extensive screening criteria may have excluded potentially valuable contributions that did not precisely align with the predefined parameters. Examples would include mixed‐method studies and further analysis of the qualitative data provided. Consequently, this limitation underscores the importance of recognizing the potential trade‐off between inclusivity and precision in study selection processes, prompting a careful consideration of the balance between thorough screening and the scope of study inclusion. Future research endeavours in this domain may benefit from a nuanced approach to screening criteria to strike an optimal balance between the depth and breadth of study inclusion.

## 7 Conclusion

The accessibility and availability of dental care providers proficient in catering to children with disabilities emerge as crucial elements in oral healthcare. It is crucial to provide education and access to relevant information for both professionals and parents or caregivers to enhance essential oral health literacy and ensure adequate care for this important population. Establishing holistic care and forming multidisciplinary teams play a vital role in delivering comprehensive care tailored to the specific needs of children with various disabilities. This study highlights solutions found in the existing literature and proposes a multidisciplinary care model with the potential to elevate the oral health experience for both parents and children facing disabilities.

## Author Contributions

**Shiamaa Al‐Mashhadani:** conceptualization, methodology, writing–original draft, writing–review and editing, formal analysis. **Mona Nasser:** writing–review and editing, validation, supervision, conceptualization. **Anas lsalami:** writing–review and editing, methodology, investigation. **Lorna Burns:** formal analysis, supervision, methodology, validation, writing–review and editing. **Martha Paisi:** writing–review and editing, validation, supervision.

## Ethics Statement

Ethical approval was not required for this review as data used for analysis were extracted from published studies.

## Conflicts of Interest

The authors declare no conflicts of interest.

## Data Availability

Data sharing is not applicable to this article as no new data were created or analysed in this study and no secondary data analysis was undertaken.

## Appendix A Search Strategies

Database:PsycINFO

**Table:**

(MAINSUBJECT.EXACT.EXPLODE(‘Disability’) OR ‘disabilit*’ OR ‘disabl*’) OR ab(disabl* OR ‘disabilit*’ OR ‘disabl*’)) AND ((MAINSUBJECT.EXACT.EXPLODE(‘OralHealth’) OR MAINSUBJECT.EXACT.EXPLODE(‘Dental Treatment’) OR MAINSUBJECT.EXACT(‘Dental Health’) OR MAINSUBJECT.EXACT(‘Dentists’)) OR (ti(oral health OR oral hygiene OR oral care) OR ab(oral health OR oral hygiene OR oral care)) OR (ti(tooth health* OR tooth hygiene OR tooth care OR tooth brush* OR tooth floss* OR toothbrush* OR teeth health* OR teeth hygiene OR teeth care OR dental OR dentist*) OR ab(tooth health* OR tooth hygiene OR tooth care OR tooth brush* OR tooth floss* OR toothbrush* OR teeth health* OR teeth hygiene OR teeth care or dental OR dentist*))) 88

Database: EMBASE

**Table:**

1	exp disability/	74,504
2	disabl*.ab,kw,ti.	68,550
3	disabilt*.ab,kw,ti.	3166
4	disable*.ab,kw,ti.	86,826
5	1 or 2 or 3 or 4	4094
6	dental health/	26,341
7	dental procedure/	220,895
8	exp tooth disease/	25,759
9	exp dentist/	48,830
10	(oral adj3 (health* or hygiene or care)).ab,kw,ti.	228,719
11	dental.ab,kw,ti.	8183
12	((tooth adj3 (health* or hygiene or care or brush* or floss*)) or toothbrush*).ab,kw,ti.	4246
13	(teeth adj3 (health* or hygiene or care or brush* or floss*)).ab,kw,ti.	74,111
14	dentist*.ab,kw,ti.	439,242
15	or/6‐14	75,007
16	5 and 15	804

Database:MEDLINE

**Table:**

1	exp disability/	17,243
2	disabl*.ab,kw,ti.	13,093
3	disabilt*.ab,kw,ti.	21,609
4	disable*.ab,kw,ti.	4127
5	1 or 2 or 3 or 4	175,921
6	dental health/	18,313
7	dental procedure/	44,013
8	exp tooth disease/	225,529
9	exp dentist/	7995
10	(oral adj3 (health* or hygiene or care)).ab,kw,ti.	3806
11	dental.ab,kw,ti.	77,372
12	((tooth adj3 (health* or hygiene or care or brush* or floss*)) or toothbrush*).ab,kw,ti.	414,449
13	(teeth adj3 (health* or hygiene or care or brush* or floss*)).ab,kw,ti.	4004
14	dentist*.ab,kw,ti.	80,664
15	or/6‐14	549
16	5 and 15	494

Database: CINAHL

**Table:**

S1	(MH ‘disabled‐Not Otherwise Specified’)) OR TI (disab* or Disabl* or ‘disabled’ or disabled) OR AB (disabl* or Disable* or ‘disabled’ or disable) OR SU (Disab* or Disabl* or ‘disabled’ or disable)	37,553
S2	(MH ‘Oral Health’)	12,752
S3	(MH ‘Oral Hygiene’)	5937
S4	(MH ‘Dental Care + ’)	18,517
S5	(MH ‘Dental Health Services’)	1424
S6	(MH ‘Tooth Diseases + ’)	35,558
S7	(MH ‘Dentists’)	10,488
S8	TI ((oral N3 (health* or hygiene or care))) OR AB ((oral N3 (health* or hygiene or care))) OR SU ((oral N3 (health* or hygiene or care)))	27,688
S9	TI dental or dentist* OR AB dental or dentist* OR SU dental or dentist*	136,992
S10	TI (((tooth N3 (health* or hygiene or care or brush* or floss*)) or toothbrush*)) OR AB (((tooth N3 (health* or hygiene or care or brush* or floss*)) or toothbrush*)) OR SU (((tooth N3 (health* or hygiene or care or brush* or floss*)) or toothbrush*))	5669
S11	TI ((teeth N3 (health* or hygiene or care or brush* or floss*))) OR AB ((teeth N3 (health* or hygiene or care or brush* or floss*))) OR SU ((teeth N3 (health* or hygiene or care or brush* or floss*)))	2398
S12	S2 OR S3 OR S4 OR S5 OR S6 OR S7 OR S8 OR S9 OR S10 OR S11	154,615
S13	S1 AND S12	377

Database: Dentistry and Oral Sciences Source

**Table:**

S1	DE ‘disabled’ OR DE ‘disablity’ OR DE ‘disablity in adolescence’ OR DE ‘disability in adults’ OR DE ‘disability in children’	182
S2	DE ‘disable child’	5
S3	TI (Disabl* or disabled*) OR AB (Disabl* or disabled*) OR SU (Disabl* or Disabled*)	522
S4	S1 OR S2 OR S3	524
S5	(DE ‘ORAL health’) OR (DE ‘ORAL hygiene’)	14,805
S6	(DE ‘DENTAL care’) OR (DE ‘DENTISTS’)) OR (DE ‘DENTAL pathology + ’)	37,516
S7	TI ((oral N3 (health* or hygiene or care))) OR AB ((oral N3 (health* or hygiene or care))) OR SU ((oral N3 (health* or hygiene or care)))	35,208
S8	TI (dental or dentist*) OR AB (dental or dentist*) OR SU (dental or dentist*)	255,752
S9	TI (((tooth N3 (health* or hygiene or care or brush* or floss*)) or toothbrush*)) OR AB (((tooth N3 (health* or hygiene or care or brush* or floss*)) or toothbrush*)) OR SU (((tooth N3 (health* or hygiene or care or brush* or floss*)) or toothbrush*))	12,580
S10	TI ((teeth N3 (health* or hygiene or care or brush* or floss*))) OR AB ((teeth N3 (health* or hygiene or care or brush* or floss*))) OR SU ((teeth N3 (health* or hygiene or care or brush* or floss*)))	9849
S11	S5 OR S6 OR S7 OR S8 OR S9 OR S10	264,853
S12	S4 AND S11	32

Database: SocINDEX

**Table:**

S1	DE ‘DISABLE’ OR DE ‘DISABILITY in adolescence’ OR DE ‘DISABLITY in adults’ OR DE ‘DISABLE in children’	1981
S2	TI (Disabl* or Disable* or ‘disability’) OR AB (Disabl* or Disable* or ‘Disability’) OR KW (Disabl* or disabile* or ‘disability’)	3827
S3	S1 OR S2	3909
S4	DE ‘DENTAL care’	624
S5	TI ((oral N3 (health* or hygiene or care))) OR AB ((oral N3 (health* or hygiene or care))) OR KW ((oral N3 (health* or hygiene or care)))	1063
S6	TI (dental or dentist*) OR AB (dental or dentist*) OR KW (dental or dentist*)	4373
S7	TI (((tooth N3 (health* or hygiene or care or brush* or floss*)) or toothbrush*)) OR AB (((tooth N3 (health* or hygiene or care or brush* or floss*)) or toothbrush*)) OR KW (((tooth N3 (health* or hygiene or care or brush* or floss*)) or toothbrush*))	208
S8	TI ((teeth N3 (health* or hygiene or care or brush* or floss*))) OR AB ((teeth N3 (health* or hygiene or care or brush* or floss*))) OR KW ((teeth N3 (health* or hygiene or care or brush* or floss*)))	148
S9	S4 OR S5 OR S6 OR S7 OR S8	4839
S10	S3 AND S9	4

Grey Literature Search

**Table:**

Organization	Web address	Search string
World Dental Federation (FDI)	| FDI (fdiworlddental.org)	Disability, oral health, oral health and disability
British Society of Special Care Dentistry	Home Page (bsdh.org)	Child or children with disability and oral health
WHO	Disability in children and adolescents must be integrated into the global health agenda (who.int)	Children with disabilities and oral health
International Association of Disability and Oral Health	iADH—A Society Aiming to Offer World Class Oral Health Care for People with Disabilities	Oral health, disabled child or children

## Appendix B Description for all studies

**Table:**

Reference	Study design	Descriptive themes
1‐Dental students' perceptions before and after attending a centre for children with special needs: A qualitative study on situated learning. Fadel, H. T., Baghlaf, K., Ben Gassem, A., Bakeer, H., Alsharif, A. T., & Kassim, S. (2020).	Qualitative semi‐structured questions of 39 fifth‐year dental students	Theme 1: Negative expectations Lack of training Organization Uncooperative children Unsuitable environment Theme 2: Positive expectations Oral health instructions for child and parent Behaviour management Tell, show, do Theme 3: Oral Problems Observed poor oral hygiene and high plaque levels due to brushing difficulties, periodontal diseases, dental caries, and bruxism
2‐Perception of dental visit pictures in children with autism spectrum disorder and their caretakers: A qualitative study. *Journal of International Society of Preventive & Community Dentistry*, 6(4), 359. Wibisono, W. L., Suharsini, M., Wiguna, T., Sudiroatmodjo, B., Budiardjo, S. B., & Auerkari, E. I. (2016).	Qualitative semi‐structured interviews with 10 autistic children aged 13−17 years, Qualitative open‐ended questionnaire with two parents, and two teachers	Facilitators 1‐Visual instructions in the form of Two dimensional (written words, line drawing, picture) or Three dimensional (gesture or expression) are better interpreted than verbal instructions in Autistic children 2‐Visualization could help in familiarizing the child with the procedure before the dental visit
3‐Mothers' perceptions concerning oral health of children and adolescents with Down syndrome: a qualitative approach. *European Journal of Paediatric Dentistry*, 11(1), 27‐30. Oliveira, A. C., Pordeus, I. A., Luz, C. L., & Paiva, S. M. (2010).	Qualitative in‐depth interviews of 19 mothers	1‐Financial constraints 2‐Time to take the child to the dentist as they were busy with other medical appointments 3‐Access to healthcare referral services
4‐Oral health–not a priority issue AG rounded theory analysis of barriers for young patients with disabilities to receive oral healthcare on the same premise as others. *European Journal of Oral Sciences*, 120(3), 232‐238. Klingberg, G., & Hallberg, U. (2012). 232‐238.	Qualitative in‐depth interviews with 65 informants (14 parents, 18 dental healthcare professionals, 17 medical healthcare professionals and 16 individuals with disabilities)	Defective knowledge about the importance of oral health Limited ability to focus on oral health Uncertainty in treating the unknown
5‐Strategies for success: A qualitative study of caregiver and dentist approaches to improving oral care for children with autism. *Pediatric Dentistry*, 41(1), 4E‐12E. Stein Duker, L. I., Floríndez, L. I., Como, D. H., Tran, C. F., Henwood, B. F., Polido, J. C., & Cermak, S. A. (2019).	Focus groups were conducted with two groups of parents of children with ASD and two groups of dentists treating children with ASD	Parents focus group Theme 1: what makes a good dentist? Theme 2: flexibility and techniques—strategies used by the dentist. Theme 3: preparation—strategies for parents and caregivers of children with ASD Dentist interviews: Theme 1: Parents know best Theme 2: Continued practice and training Theme 3: Flexibility Theme 4: A network of colleagues. Areas of overlap between the parents and dental providers: the importance of preparation, the necessity of flexibility and creativity, and the value of collaboration
6‐Barriers to oral health promotion in the Republic of Ireland Owens, J. (2011). Barriers to oral health promotion in the Republic of Ireland. *Scandinavian Journal of Public Health*, 39(6_suppl), 93‐97.	Focus group of 15 parents Qualitative interviews for 18 non‐dental professionals	Theme 1: Budget constraints Theme 2: The daily demands of caring for a child with disabilities are tied to a day‐to‐day existence
7‐Qualitative study on barriers and coping strategies for dental care in autistic children: Parents' perspective. International *Journal of Paediatric Dentistry*, 33(2), 203‐215. Junnarkar, V. S., Tong, H. J., Hanna, K. M. B., Aishworiya, R., & Duggal, M. (2023).	Qualitative semi‐structured interviews for 23 parents of autistic children and a focus group discussion were used for 23 parents of autistic children	Theme 1: Toothbrushing challenges and use of non‐optimally fluoridated toothpaste are related to sensory, physical or parental dental knowledge issues Theme 2: Delayed dental visits stem from parental concerns about managing the child, personal attitudes towards dentistry, lack of information on special needs dentists and perceived high cost of dental treatment Theme 3: Parental Involvement and input for dental visits is essential to overcome challenges for dental visits
8‐Preventive oral healthcare use for children with special healthcare needs aged 6 through 12 years enroled in Medicaid: A mixed methods study. *The Journal of the American Dental Association*, 152(10), 800‐812. Lang, C., Kerr, D., & Chi, D. L. (2021).	Qualitative semi‐structured interviews with 21 participants of community stakeholders	Theme 1: Caries risk depends on a child's specific health condition caries complicates overall health Theme 2: Having a special need creates a bigger barrier to care Theme 3: Legislation alone is not going to make much of a dent Theme 3: Improvements across all fronts are needed to promote the oral health of CSHCN enroled in Medicaid
9‐ Accessibility to the oral health of people with Intellectual Disabilities from the caregiver's perspective: a qualitative evaluation. *Rev Bras Odontol*, 75, e1107. Alves, F. R., Alves, N. S., Gavina, V. P., Silveira, F. M., do Espírito Santo, T. M., & Assaf, A. V. (2018).	Qualitative semi‐structured interviews of 55 caregivers of people with ID	Theme 1: Lack of structure and organization of the service Theme 2: Deficient training of professionals for attendance, among others. Theme 3: The healthcare within a practice is weak on health promotion were other factors that compromised the access of people with ID
10‐Medical healthcare professionals' assessments of oral health needs in children with disabilities: a qualitative study. *European Journal of Oral Sciences*, 113(5), 363‐368. Hallberg, U., & Klingberg, G. (2005).	In‐depth interviews focusing on orofacial function were carried out with 17 medical healthcare employees	Theme 1: Oral healthcare assessment was not on the agenda of medical healthcare professionals, but was instead viewed as the responsibility of parents or dentists
11‐Perspectives of the public dental workforce on the dental management of people with special needs. *Australian Dental Journal*, 66(3), 304‐313. Lim, M. A. W. T., Liberali, S. A. C., Calache, H., Parashos, P., & Borromeo, G. L. (2021).	Qualitative interviews and focus groups of oral health professionals working in the Australian public dental system	Theme 1: Difficulties associated with treating patients with special needs Theme 2: (Different proposed supports suggested by clinicians
12‐Dental health professionals' treatment of children with disabilities: a qualitative study. *Acta Odontologica Scandinavica*, 62(6), 319‐327. Hallberg, U., Strandmark, M., & Klingberg, G. (2004).	In‐depth interviews focusing on orofacial function, and carried out with 18 informants (dentists, dental hygienists, dental assistants)	Theme 1: Variability in treatment with the dimension's professional uncertainty and professional commitment
